# Inflammatory and lipemic response to red meat intake in women with and without Rheumatoid Arthritis: a single meal study within a randomized controlled trial

**DOI:** 10.1186/s40795-025-01055-9

**Published:** 2025-04-11

**Authors:** Torsten Sällström, Linnea Bärebring, Erik Hulander, Inger Gjertsson, Anna Winkvist, Helen M. Lindqvist

**Affiliations:** 1 Department of Internal Medicine and Clinical Nutrition, Institute of Medicine, Sahlgrenska Academy, University of Gothenburg, PO Box 459, Gothenburg, 405 30 Sweden; 2 Department of Rheumatology and Inflammation Research, Institute of Medicine, Sahlgrenska Academy, University of Gothenburg, Gothenburg, Sweden

**Keywords:** Rheumatoid arthritis, Postprandial inflammation, Postprandial lipaemia, Interleukin- 6, Red meat

## Abstract

**Background:**

The risk of atherosclerotic cardiovascular disease (ASCVD) is increased in Rheumatoid Arthritis (RA). Previous research has suggested that lipid metabolism is altered in RA, but research under postprandial conditions is scarce. The aim of this study was to investigate whether women with RA have a different lipemic and inflammatory response to a mixed meal containing red meat compared to women without RA.

**Methods:**

Twenty-two women with RA, with modest disease activity, and 22 women without RA matched for age and body mass index (BMI) at the group level consumed a hamburger meal containing ca. 700 kcal (53 E% from fat, 27 E% from carbohydrate). Venous blood was sampled in the fasted state and after 30 min, 1, 2, 3 and 5 h and analysed for lipid species using nuclear magnetic resonance spectroscopy. Postprandial inflammation was measured by interleukin- 6 (IL- 6). The postprandial lipid response was calculated as the incremental area under the curve minimal value, and serial measurements were analysed by repeated measures analysis of variance. Lipid and inflammatory responses were compared by linear regression analysis, adjusted for age, BMI, physical activity, and baseline plasma concentration.

**Results:**

Plasma concentrations of IL- 6, triglycerides (TGs) and very low-density lipoprotein (VLDL) particles increased significantly after the meal compared to baseline within both groups, but no differences were observed between groups. However, the women with RA had a less pronounced response in cholesterol carried in VLDL particles (*p* = 0.03) and in TGs in the subfraction of VLDL particles with highest density (*p* = 0.03). No association was found between the response in TGs and IL- 6.

**Conclusion:**

This study does not provide compelling evidence for any difference in the lipemic or inflammatory response in women with RA compared with age- and BMI-matched women without RA following ingestion of a mixed, high-fat meal containing red meat. The modest disease activity in women with RA should be considered when interpreting these findings. Subtle group differences found in the lipids carried by VLDL particles warrant further investigation.

**Trial registration:**

The PIRA (Postprandial Inflammation in Rheumatoid Arthritis) trial was registered 2020–01 - 28 at Clinicaltrials.gov (NCT04247009).

**Supplementary Information:**

The online version contains supplementary material available at 10.1186/s40795-025-01055-9.

## Background

Rheumatoid arthritis (RA) is a systemic, autoimmune disease estimated to affect between 0.5–1.0% of the population worldwide, with women being disproportionally afflicted [1, 2]. RA is characterised by inflammation of peripheral joints with a detrimental impact on biomechanical function and quality of life [3]. Chronic inflammation predisposes patients to a wide range of extra-articular comorbidities. Notably, individuals with RA suffer an increased risk of atherosclerotic cardiovascular disease (ASCVD) beyond what can be explained by conventional risk factors [4, 5].

Dyslipidaemia, particularly elevated levels of cholesterol carried in the low-density lipoprotein (LDL) fraction, is widely recognised as a major and causal risk factor for ASCVD [6]. Recent years have seen a surging interest in the role of triglyceride-rich lipoproteins (TRLs), comprising both hepatic very low-density lipoproteins (VLDLs) and intestinally derived chylomicrons (CMs) [7]. In clinical practice, the assessment of blood lipids has traditionally been performed in the fasting state [8]. However, as a typical meal pattern consists of several meals and snacks consumed during the day, blood lipids are rarely at fasting levels for more than a few hours in the early morning [9, 10]. Elevated levels of blood triglycerides after a meal (i.e. the postprandial period) is known as postprandial hyperlipidaemia which is an independent ASCVD risk factor [11, 12].

A large number of studies have reported irregularities in the fasting blood lipid profile in persons with RA [13–15], leading to the assumption that the disease might be associated with alterations in lipid metabolism [16]. However, there is a scarcity of research on the postprandial lipemic response in this population, with only one previously published meal trial to date [17]. In that study, significantly higher concentrations of apoB- 48 were observed after a meal in individuals with RA compared with healthy controls, suggesting possible alterations in either intestinal synthesis of CMs, hepatic clearance of their remnants, or both [17].

The ingestion of a high-fat meal generally elicits some degree of immune activation [18, 19]. Although the clinical relevance of this transient inflammatory response remains unclear, it is well established that persistently elevated levels of circulating proinflammatory molecules contribute to the development and acceleration of atherosclerosis [20]. IL- 6 is the most frequently assessed marker of postprandial inflammation, with most – but not all – studies reporting significantly increased plasma concentrations after a high-fat meal [21]. As patients with RA have a chronic inflammatory condition, postprandial inflammatory response may differ in RA compared to a healthy population, but this has to our knowledge never been assessed.

A substantial proportion of patients with RA report subjective benefits when avoiding red meat [22, 23] but objective data are lacking. The evidence for a link between red meat intake, inflammation and serum lipid profile remains limited and inconclusive also in the general population [24], despite correlation between consumption of red meat and markers of inflammation in large cohorts [25, 26]. Moreover, a diet rich in red meat has been identified as a significant contributor to cardiovascular disease in high- and middle income countries [27], but results from randomized controlled trials are inconclusive with regard to effects on serum lipid profile [28].

To summarise, individuals with RA have a substantially increased risk of ASCVD, and a growing body of research emphasises the role of postprandial TRLs as a cardiovascular risk factor. Further, postprandial inflammation is a poorly understood metabolic phenomenon in general and in patients with RA. Previous research has suggested altered lipid metabolism in RA, but research is scarce. The aim of this study was to investigate whether women with RA exhibit a different lipemic and inflammatory response to a mixed meal containing red meat compared with that of women without RA.

## Methods

Postprandial Inflammation in Rheumatoid Arthritis (PIRA) (NCT04247009) was a randomized controlled clinical trial, with the main objective of investigating the postprandial inflammatory and metabolic response to three meals with different protein sources in women with RA [29]. A control group consisting of women without rheumatic disease also consumed one of the test meals, based on red meat. In the present paper, the postprandial responses to the red meat-containing meal in the two groups are compared. The study was initiated in February 2020, halted shortly thereafter due to the COVID- 19 pandemic, resumed in August 2021, and concluded in November of the same year.

### Recruitment

Presumptive participants (*n* = 934) were identified through the Swedish Rheumatology Quality Registry and invited by postal letter. Eligible participants were women residing in the region of Västra Götaland in southwestern Sweden and meeting the diagnostic criteria for RA as defined by the American College of Rheumatology (ACR) and European League Against Rheumatism (EULAR) 2010 [30]. Further inclusion criteria were ≥ 2 years since diagnosis, stable Disease Modifying Anti-Rheumatic Drug (DMARD) treatment for the last three months, a body mass index (BMI) of 18.5–30 kg/m^2^ and age 20–70 years. Exclusion criteria were treatment with IL- 6 inhibitors, lipid lowering medication, cancer diagnosis, inflammatory bowel disease, celiac disease, diabetes, pregnancy or lactation, a haemoglobin concentration of < 100 g/l, smoking and allergy or intolerance to any of the ingredients of the test meals. Women without RA were recruited to the control group through advertisements on social media, on public noticeboards and by word of mouth. Inclusion criteria for controls were female sex, aged 20–70 years, BMI 18.5–30 kg/m^2^, considering oneself healthy and agreeing to consume red meat. The same exclusion criteria as for participants with RA were applied. The initial plan was to match participants pairwise based on age, BMI and reported physical activity level. This proved unfeasible, and matching was therefore performed at the group level by selecting the women without RA that created the most similar group mean, median and min–max range as the women with RA with respect to BMI and age.

The PIRA study was approved by the Swedish Ethical Review Authority (Dnr 2019–05242). Written informed consent was obtained prior to enrolment. All procedures were conducted according to the Declaration of Helsinki. The reporting of this study adheres to the Consolidated Standards of Reporting Trials (CONSORT) guidelines.

### Screening

At the screening visit, participants’ height was measured without shoes to the nearest 1.0 cm and waist and hip circumference to the nearest 0.5 cm. Participants were asked to void their bladder and were subsequently weighed on a digital scale (Tanita MC- 180 MA, Tanita, Tokyo, Japan) in a standard hospital gown. Body weight was recorded to the nearest 0.5 kg. Non fasting blood samples were collected, and high-sensitivity C-reactive protein (hsCRP), erythrocyte sedimentation rate (ESR) and glycated haemoglobin (HbA1c) were analysed in fresh samples with standard clinical assays at Sahlgrenska University Hospital. Disease activity among women with RA was assessed by trained research nurses at the Clinical Rheumatology Research Centre, Sahlgrenska University Hospital, Gothenburg, according to Disease Activity Score (DAS)28-ESR—a composite score comprising a physical examination of 28 joints, ESR and perceived healthiness on a visual analogue scale. Based on the DAS28-ESR score, disease activity was categorised as in remission (< 2.6), low (2.6 to ≤ 3.2), moderate (> 3.2 to ≤ 5.1) and high (> 5.1). A self-administered questionnaire provided information about habitual intake (food frequency), supplement use, education level and physical activity level at work and during leisure time. The physical activity level was calculated to resemble the Cambridge Index, which ranges from 1 (= inactive) to 4 (= active) [31]. Dietary quality index was assessed based on food frequency and an index ranging from 1 to 12 was constructed, as previously described by the Swedish Food Agency [32].

### Test meal

The design of the PIRA study has been described in detail elsewhere [33]. Briefly, participants arrived at the study centre at the Department of Internal Medicine and Clinical Nutrition, University of Gothenburg, in the morning after an overnight fast. They had been instructed not to ingest alcohol or caffeine and not to participate in any vigorous physical exercise after 10 p.m. the night before. A hamburger meal was served, which the participants were asked to consume within 20 min. The meal consisted of two minced meat patties on white toast with lettuce, cucumber, tomato, and a plant-based dressing and was accompanied by one glass of water. The patties contained about 130 g of minced meat (60% beef, 40% pork), breadcrumbs and eggs (Supplemental table S.1) [See Additional file 1]. Each meal provided 692 kcal, 40.8 g fat (53.1 E%), 46.8 g carbohydrates (27.1 E%) and 34.7 g protein (20.1 E%) (Table 1). The nutritional content of the minced meat patties was analysed by an accredited external laboratory (Eurofins Food & Feed Testing, Lidköping, Sweden). For the remaining ingredients, nutritional content was calculated based on the values declared by the producer where applicable or using generic values from the Swedish Food Authority’s food composition database (version 2017.12.15).

**Table 1:** Nutrient composition of the test meal

	Per meal	E%
Energy, *kcal*	690	
Protein,* g*	35	20
Fat, *g*	41	53
SFA, *g*	10	12
Carbohydrates^a^, *g*	47	27

E%, energy percentage; *Kcal* kilocalories, *SFA* saturated fatty acids

^a^Glycaemic carbohydrate, fibre not included

### Blood collection and biochemical analyses

Immediately before consuming the test meal, a catheter was inserted in an antecubital vein, and fasting blood samples were collected. Blood was then drawn at 30, 60, 120, 180 and 300 min after completion of the meal into serum separator vacutainer tubes (Becton Dickinson, Meylan Cedex, France). The samples were inverted 8 times, left at room temperature for 30 min and then refrigerated for 30 min before being centrifuged for 10 min at 3800 rpm. After aliquotation, the samples were frozen at − 20 °C and subsequently stored at − 80 °C until analysis. Highly sensitive IL- 6 was analysed at fasting and at 300 min postprandially with Electrochemiluminescence Immunoassay (ECLIA) on a Cobas (Roche Diagnostics, Basel, Switzerland) by the Sahlgrenska University Hospital (intra-assay coefficient of variation: 1.99% at 30 pg/ml [Personal email communication with the Department of Clinical Chemistry, Sahlgrenska University Hospital Östra, 23 Jan 2020]) Quantification of blood lipids and lipoproteins was performed by nuclear magnetic resonance (NMR) spectrometry at the Swedish NMR-centre, University of Gothenburg. Measurements were performed on a Bruker 600 MHz Avance III spectrometer (Bruker, Billerica, MA, USA) according to the protocol for In Vitro Diagnostic Research (IVDr). The procedure has been described in detail elsewhere [34]. A total of 21 lipid parameters were assessed, including triglycerides (TGs), total cholesterol (TC), high-density lipoprotein cholesterol (HDL-C), low-density lipoprotein cholesterol (LDL-C), very low-density lipoprotein cholesterol (VLDL-C) and apolipoprotein B- 100 (apoB- 100) as well as particle numbers and TG-content of lipoproteins within different density ranges. Classes and subfractions of lipoproteins were categorised according to the specific B.I.-LISA model [35]. According to this model, lipoprotein particles with a density of 0.950–1.006 g/ml were classified as VLDL, and this class was further divided into five subfractions (VLDL- 1 – VLDL- 5) according to increasing density [36]. Units were converted from mg/dl to mmol/l using a factor of 0.02586 for cholesterol and 0.01129 for TG [37].

### Statistical analyses

Postprandial changes in plasma IL- 6 concentrations were expressed both in absolute terms by subtracting baseline values from those at 300 min after the test meal and in relative terms as the percentage increase. Temporal changes in plasma lipid concentrations were analysed with a two-way repeated measures analysis of variance (RM-ANOVA) with group (with/without RA) as between-subjects factor and the plasma concentrations measured at each time point as within-subjects variables. When the assumption of sphericity was violated, Greenhouse‒Geisser correction was applied. Missing values for eight single data points (seven in the intermediate-density lipoprotein [IDL] range and one in the VLDL- 1 range) were missing and imputed. Imputation was performed by inserting the mean of the two adjacent values when available or by adding the group mean percentual change to the penultimate value when the missing value was last in a time series. Postprandial lipid response curves were plotted, and the incremental area under the curve with the lowest measured concentration as baseline (AUC_min_) was approximated using the trapezoidal rule by equation [A] as previously described [38, 39].

A $${AUC}_{min}=\sum \frac{1}{2}(\left({y}_{i}-{y}_{min}\right)+\left({y}_{i+1}-{y}_{min}\right))({t}_{i+1}-{t}_{i})$$

where *y* denotes the concentration of a given lipid at timepoint *i* in mmol/l, *y*_*min*_ is the lowest registered concentration and *t* is the time elapsed in minutes at timepoint *i*.

Group differences in postprandial response were analysed by multivariable linear regression analysis adjusted for age, BMI, physical activity level and baseline plasma concentration. The same model was used to analyse the association between AUC_min_ for TGs and percent change in concentrations of IL- 6. Variables were transformed by log10 or cubic root as needed to satisfy assumptions. For the regression analyses involving changes in IL- 6, three outliers from the RA group had to be excluded to comply with assumptions regarding homoscedasticity.

Continuous variables were reported as medians with interquartile ranges (IQRs, 25 th and 75 th percentiles unless otherwise indicated). Normality was assessed with Kolmogorov-Smirnoff’s test. Comparisons of means between groups were performed using independent t-tests when the assumption of normality was met and otherwise by the Mann‒Whitney U test. Wilcoxon signed rank test was used for comparing IL- 6-levels within groups before and after the meal. Categorical variables were analysed by the χ^2^-test. Spearman’s rank order correlations (denoted by the coefficient rho, *ρ*) were performed to explore the relationship between variables. AUC_min_ was calculated in Excel (version 2302, Microsoft Corporation, WA, USA). All statistical analyses were performed using IBM SPSS Statistics for Windows version 29 (IBM Corp., Armonk, N.Y., USA). The two-tailed significance level was set to *p* < 0.05.

### Power calculations

Sample size for the PIRA-trial was calculated based on the primary objective, which was to compare the response in IL- 6 to three meals of different composition among women with RA, and has been described in detail elsewhere [33]. In that context, we aimed to recruit 30 patients with RA. In the current work, 22 patients and 22 matched controls are included. Although our study was not originally powered to detect between-group differences, the achieved sample size exceeds those of previous studies that have detected group differences in TGs [40, 41] and conforms with recommendations for studies of postprandial lipid metabolism [42]. The sample size is also similar to other studies that have detected postprandial changes in IL- 6 within groups [21], indicating that the sample size is appropriate for this purpose. Few studies with similar sample size [43] have reported on group differences in postprandial changes in IL- 6, making it difficult to assess its adequacy in this respect.

## Results

### Subjects

**Fig. 1:**
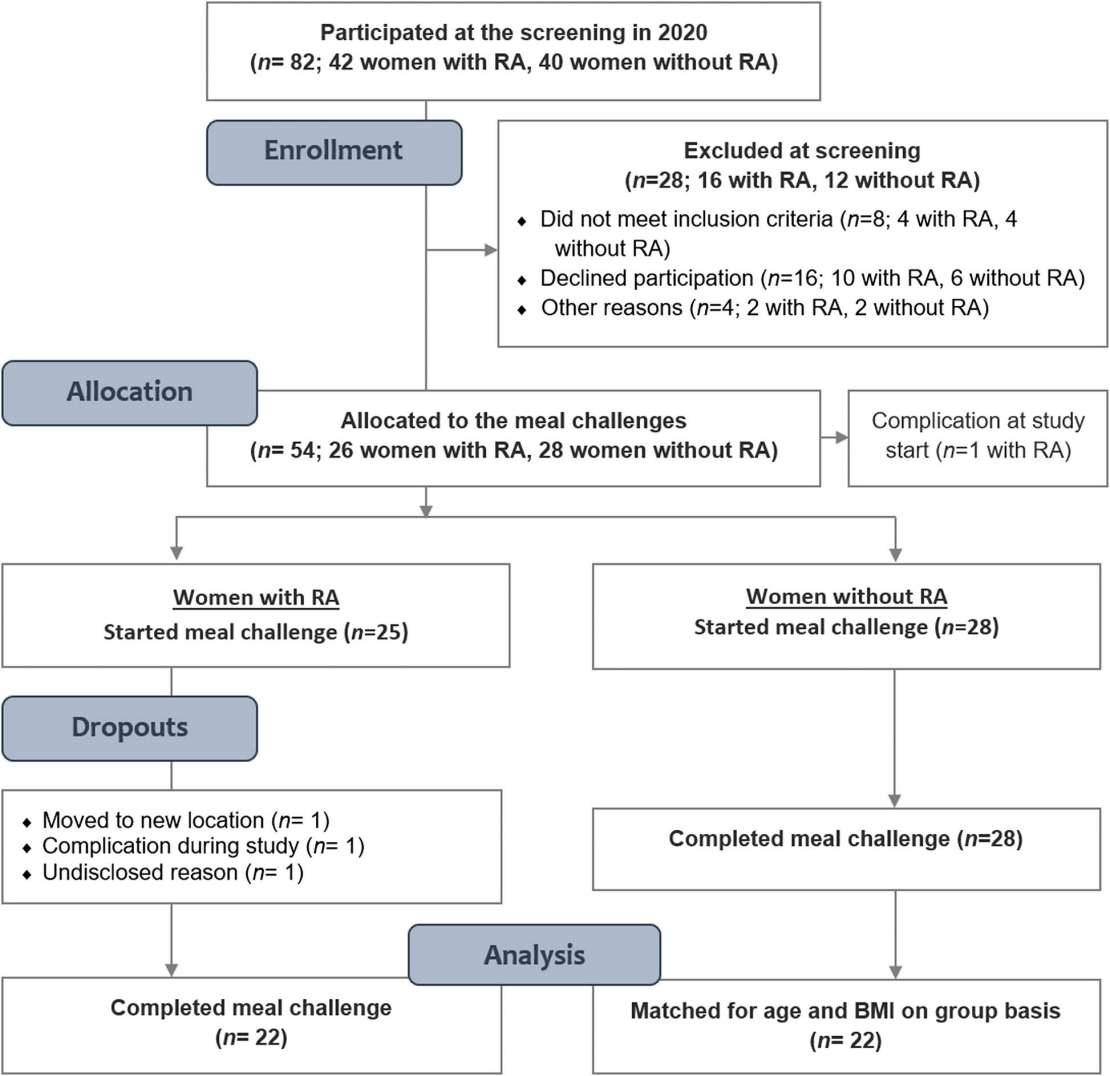
CONSORT flowchart of subject recruitment for the PIRA study

Forty-two women with RA and 40 without RA attended the PIRA screening visit in 2020 (Fig. 1). When the study resumed in 2021 after a pause due to the COVID- 19 pandemic, ten women with RA and six without RA declined to participate. One further individual without RA was asked not to continue due to low plasma haemoglobin levels and another due to deviations in the matching criteria. The extended gap between the screening visit and test meal for some of the participants resulted in one person from each group no longer meeting the inclusion criterion for BMI (by less than 1 kg/m^2^) at the time of the test meal and one person in the RA group having surpassed the age limit (by less than 1 year). They were nonetheless retained in the trial. After initial exclusions and dropouts, 54 participants commenced the meal challenges. Twenty-two women with RA and 28 without RA completed the red meat-containing test meal. Out of the 28 without RA, the 22 that created the most similar group in terms of mean age and BMI were selected to obtain matching at the group level. Hence, the present analyses include 44 subjects.

All participants resided in the greater Gothenburg area and reported having parents of European descendance and most had an academic degree (Table 2). The median age was about 65 years and all except three in each group had reached menopause. More women with RA reported leading a physically active life (45.5% with RA; 13.6% without RA, *p* = 0.057). No participants at all had a habitual diet with a high diet quality index (9–12 points), but the majority had a fair diet quality index (5–8 points) (Table 2). All participants had an HbA1c within the normal range, although it was significantly higher among women without RA (35 vs. 32 mmol/mmol, *p* = 0.020). In terms of disease activity markers in the RA group, plasma hsCRP was 1.3 mg/l (0.5–3.6), and they had a median DAS28-ESR score of 2.54 (1.94–4.1). A majority (*n* = 12; 55%) were classified as being in remission, while for the remainder, disease activity was classified as low in two (9%) and moderate in eight (36%) individuals. Eighteen of the women with RA (82%) were treated with the disease-modifying antirheumatic drug methotrexate. Vitamin B, vitamin D, and omega- 3 supplementation was reported by 5, 7, and 3 women with RA versus 1, 5 and 1 women without RA.

**Table 2:** Demographic and anthropometric characteristics of the participants

	Women with RA (*n* = 22)			Women without RA (*n* = 22)			
	Median	IQR	Min–Max	Median	IQR	Min–Max	*p*^*1*^
Age, *years*	66	59–69	45–71	64	58–67	45–70	0.372
Height, *cm*	167.5	162.8–170.3	155.0–182.0	167.0	165.0–172.8	158.0–179.5	0.437
Weight, *kg*	69.3	60.9–76.0	48.1–87.3	68.4	64.1–74.6	51.7–89.6	0.851
BMI, *kg/m*^*2*^	24.6	22.7–27.1	18.6–30.9	24.1	22.7–26.2	20.7–31.4	0.606
Waist circumference, *cm*	85.5	77.0–91.5	68.0–102.0	82.0	78.8–26.2	69.0–101.0	0.934
Waist/hip-ratio	0.82	0.78–0.88	0.49–0.99	0.81	0.77–0.84	0.71–0.97	0.385
HbA1c, *mmol/mmol*	32	31–34	21–38	35	31–37	26–40	0.020
Diet quality index	5.5	5–6	3–8	5	4.8–7	2–8	0.913
Education	*n*	%		*n*	%		0.200^2^
Junior high school	1	4.5		0	0		
2 year senior high school	3	13.6		0	0		
≥ 3 year senior high school	1	4.5		4	18.2		
College/University degree	17	77.3		18	81.8		
Physical activity level	*n*	%		*n*	%		0.057^2^
Inactive	3	13.6		2	9.1		
Moderately inactive	5	22.7		13	59.1		
Moderately active	4	18.2		4	18.2		
Active	10	45.5		3	13.6		

*BMI* Body mass index, *HbA1c* Glycated haemoglobin, *IQR* Inter quartile range (q1-q3), *RA* Rheumatoid arthritis

^1^Means compared using the Mann‒Whitney U test unless otherwise indicated

^2^χ^2^ test

**Fig. 2:**
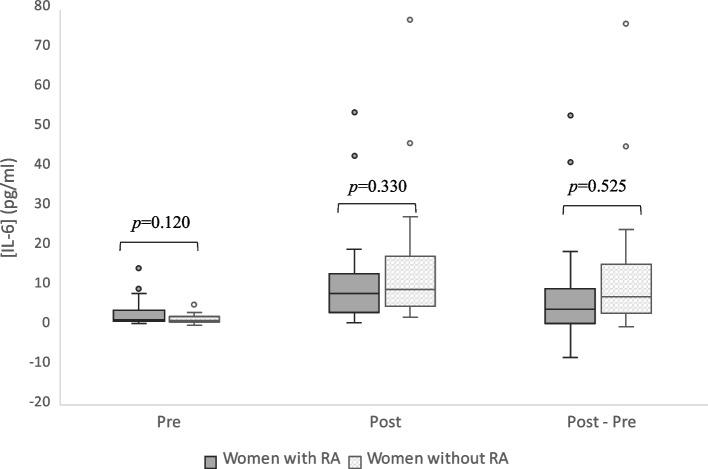
Plasma concentrations of interleukin- 6 before (Pre) and five hours after the test meal (Post). *p* values for group differences assessed by multivariable regression with baseline plasma concentration, age, body mass index and physical activity as covariates. The post minus pre analysis included 41 individuals (*n* = 19 with RA, *n* = 22 without RA) after exclusion of three outliers. The dependent variables Pre and Post meal were log10-transformed, while the post minus pre variable was cubic root transformed

Plasma concentrations of IL- 6 were similar between groups at baseline, with median values of 1.72 pg/ml (1.14–4.01) in the RA group and 1.41 pg/ml (1.08–2.42) in the women without RA (*p* = 0.120, Fig. 2). In terms of fasting levels of the lipid parameters, no difference was seen between groups (Supplemental table S.2) [See additional file 1]. Fasting TG concentrations were generally well within the normal range. Four of the women with RA and five without RA exhibited borderline elevated TG levels (≥ 1.2 mmol/l), as proposed by the European Society of Cardiology (ESC) [7, 44].

## Postprandial response

### Inflammation

At five hours after the meal, IL- 6 had increased by 4.29 pg/ml (0.63–9.35) or 181% (40–551) in the RA group and by 7.53 pg/ml (3.22–15.63) or 591% (161–1087) in the women without RA (Fig. 2). The increase was significant compared with fasting levels within both groups (*p* < 0.001), but no difference was noted between groups (*p* = 0.525 for the absolute change and *p* = 0.361 for the percent change according to the model adjusted for baseline plasma concentrations, BMI, age and physical activity). There was a substantial variation in the IL- 6 response in both groups. The maximum increase was over 80-fold in women with RA and over 60-fold in women without RA. Simultaneously, a decrease compared to baseline was noted for three (13.6%) women with and one (4.5%) without RA at five hours after the meal.

### Lipids and apolipoproteins

**Fig. 3:**
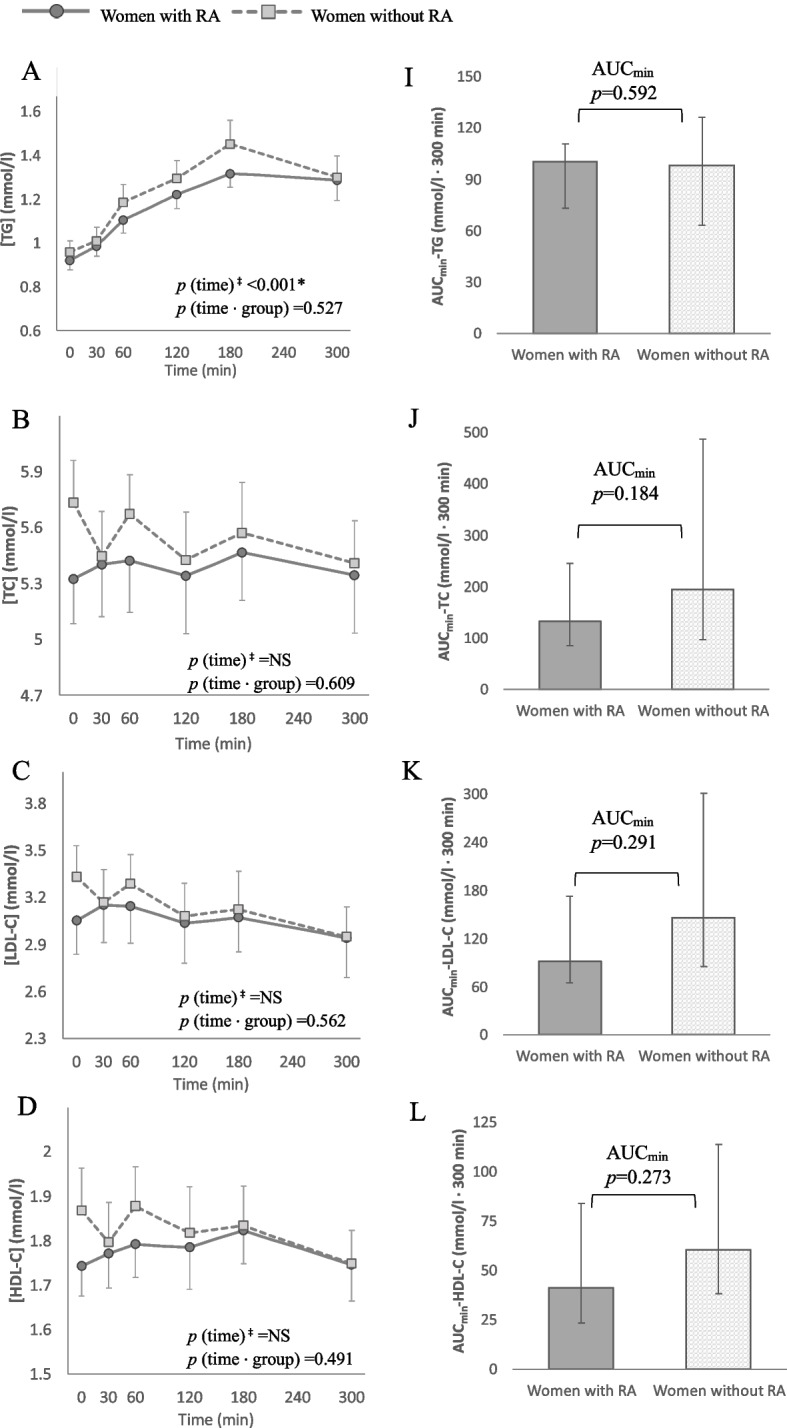
Postprandial response curves and median areas under the curve (AUCmin). Panel **A** – **D**: Postprandial response curves for triglycerides (**A**), total cholesterol (**C**), high-density lipoprotein cholesterol (**D**) and low-density lipoprotein cholesterol (**E**). Group mean values with error bars showing the standard error. *p* values from repeated-measures ANOVA of the time effect in each group and the time-by-group effect. Panel I – L: Median AUCmin for the same lipid parameters. Bars denoting the interquartile range. *p* values for the difference between groups assessed by multivariate regression analysis with fasting concentrations, age, body mass index and physical activity as covariates. Note the differences in scale on the y-axes. AUCmin; Area under the curve minimum concentration; -C, Cholesterol; HDL-C, High density lipoprotein cholesterol; LDL-C; Low density lipoprotein cholesterol; NS, Not significant; RA, Rheumatoid arthritis; TC, Total cholesterol. ‡*p* value calculated separately for each group. *Significant at α-level 0.05

**Fig. 4:**
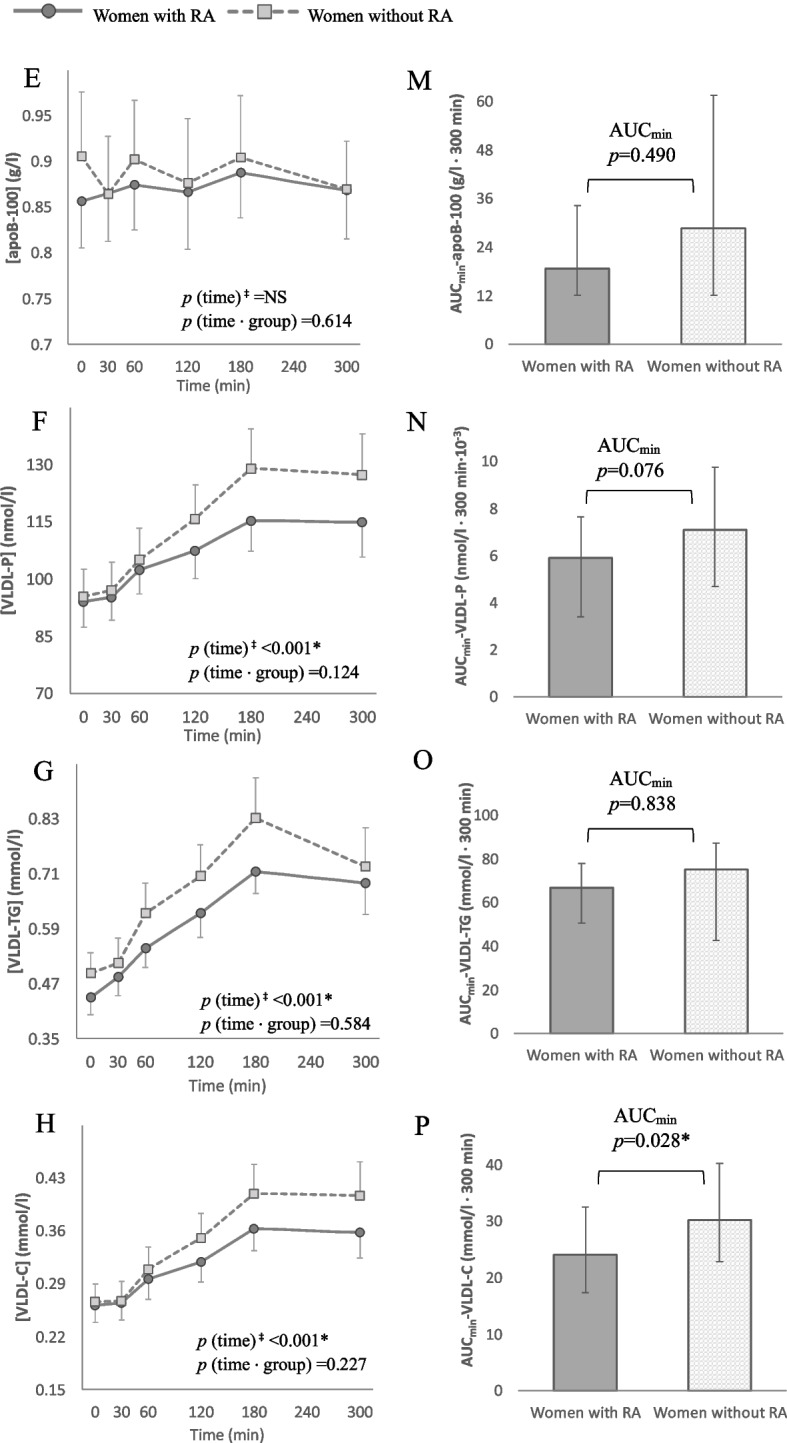
Postprandial response curves and median areas under the curve (AUCmin). Panel **E** – **H**: Postprandial response curves for apoB- 100 (**E**), VLDL particles (**F**), VLDL triglycerides (**G**) and VLDL cholesterol (**H**). Group mean values with error bars showing the standard error. *p* values from repeated-measures ANOVA of the time effect in each group and the time-by-group effect. Panel M—P: Median AUCmin for the same lipid parameters. Bars denoting the interquartile range. *p* values for the difference between groups assessed by multivariate regression analysis with fasting concentrations, age, body mass index and physical activity as covariates. Note the differences in scale on the y-axes. Apo, apolipoprotein; AUCmin; Area under the curve minimum concentration; -C, Cholesterol; NS, Not significant; -P, Particles; RA, Rheumatoid arthritis; TC, Total cholesterol; VLDL, Very low density lipoprotein. ‡*p* value calculated separately for each group. *Significant at α-level 0.05

Postprandial time-course responses along with AUC_min_ for selected lipid classes and species are presented in Figs. 3 and 4. Complete data for all lipid parameters can be found in Supplemental Table S.2 [See additional file 1]. RM-ANOVA of the TG response showed a main effect of time in both groups (*p* < 0.001) but no time-by-group interaction (*p* = 0.527). The AUC_min_ did not differ significantly between women with and without RA (*p* = 0.592). Fasting TG levels showed a moderate to high positive correlation with postprandial TG AUC_min_ (with RA; *ρ* = 0.613, *p* = 0.002, without RA; *ρ* = 0.794, *p* < 0.001) both in women with and without RA. The peak TG concentration was on average 1.42 mmol/l (1.18–1.70) in the RA group and 1.45 mmol/l (1.13–1.91) among the women without RA. The peak was typically registered at the 180-min timepoint and represented a median percentual increase of 53% from baseline in both groups. Two individuals with RA and four without RA exceeded 2 mmol/l, which has been proposed as the cut-off for elevated non-fasting TGs [8].

**Fig. 5:**
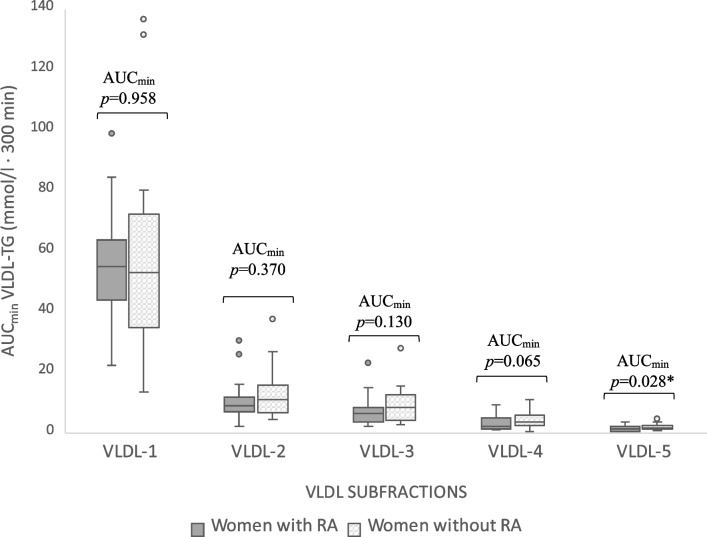
Distribution of triglycerides (TGs) between subfractions of VLDL, ordered by increasing density from 1 to 5. *p* values from group comparison of area under the curve with minimum concentrations as baseline (AUCmin), analysed by multivariable regression with baseline concentrations, age, body mass index and physical activity as covariates. All dependent variables, except VLDL- 1, were log10-transformed to satisfy test assumptions. AUCmin; Area under the curve minimum concentration; RA, Rheumatoid arthritis; TG, Triglycerides; VLDL, Very-low density lipoprotein

Plasma concentrations of TC, LDL-C, HDL-C and apoB- 100 did not change significantly in either group, nor were there any group differences in the magnitude of AUC_min_ (Figs. 3 & 4). The number of VLDL-P increased significantly within both groups (*p* < 0.001 for the main effect of time) but without a time-by-group interaction (*p* = 0.124). Further investigation into the components of the VLDL particles unveiled a similar postprandial trajectory for TGs (VLDL-TG) and cholesterol (VLDL-C) in both groups. However, the AUC_min_ for VLDL-C was significantly larger in women without RA (*p* = 0.028) (Fig. 4). Moreover, when comparing the postprandial response of TGs carried in specific subfractions of VLDL, the women without RA exhibited a larger AUC_min_ for TGs in VLDL particles with the highest density i.e. smallest size (VLDL- 5) (*p* = 0.028, Fig. 5).

### Association between response in triglycerides and interleukin- 6

**Fig. 6:**
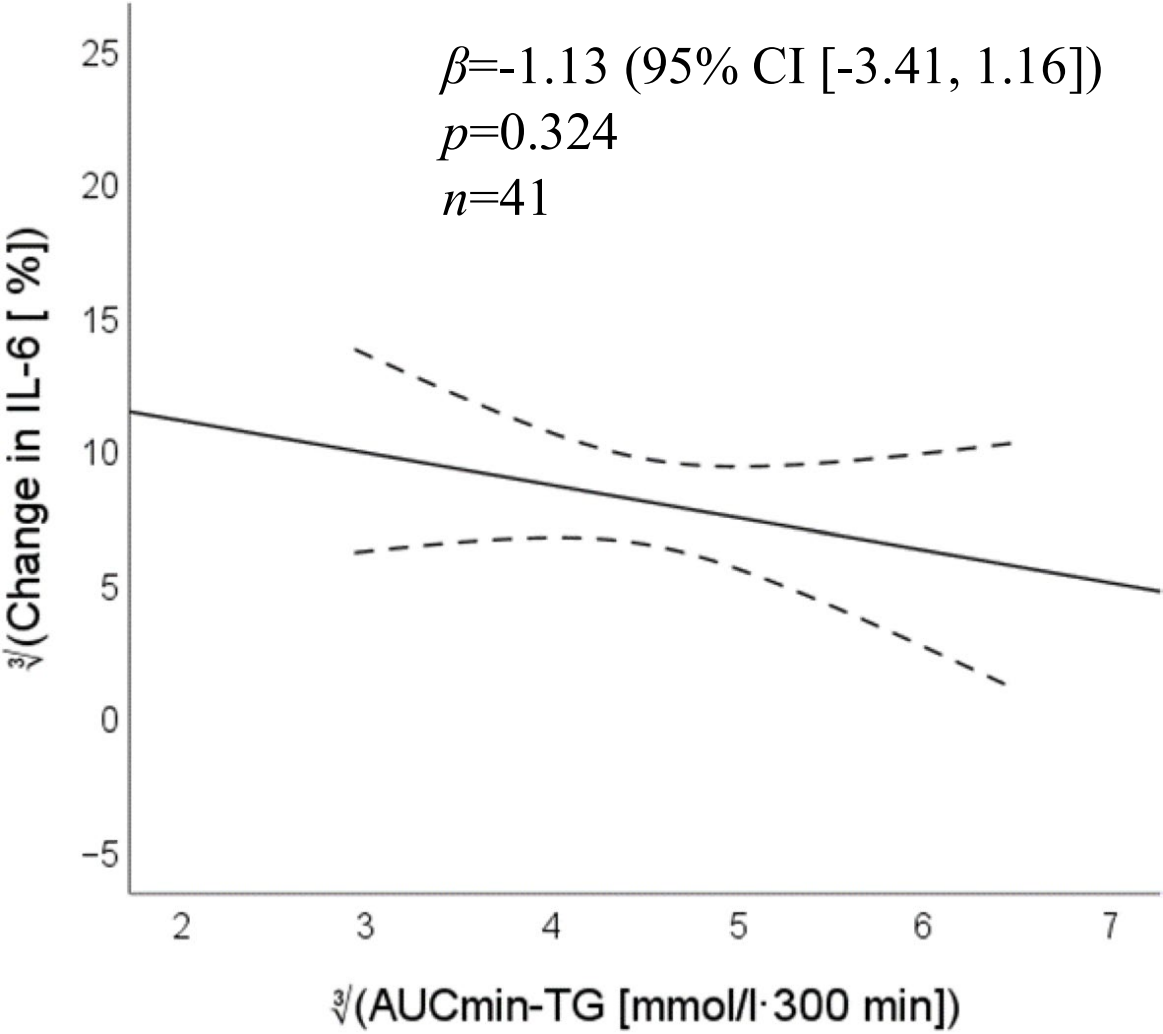
Association between postprandial triglyceride response and percent change in interleukin- 6 at five hours after the meal compared with baseline. Triglyceride response is expressed as the area under the curve minimum concentration (AUCmin). Both main variables were cubic root transformed. Dashed lines show the 95% confidence interval. β-coefficient and *p* value from the multivariable linear regression model with age, body mass index, physical activity and baseline plasma concentrations of interleukin- 6 as covariates. *n* = 41 after exclusion of three individuals in the RA group to satisfy test assumptions

There was no correlation between plasma TG and IL- 6 concentrations either at fasting (*ρ* = 0.083, *p* = 0.594) or five hours after the meal (*ρ* = − 0.111, *p* = 0.474) in the total study sample (*n* = 44). Finally, no association between AUC_min_ for TGs and percent change in IL- 6 was found (*β* = − 1.13, *p* = 0.324, Fig. 6 & Supplemental table S.4) [See Additional file 1]. No group interaction was detected (*p* = 0.347).

## Discussion

This study aimed to compare the postprandial inflammatory and lipemic responses to a high-fat meal containing red meat between women with RA and group-matched women without RA. The main finding was a largely similar postprandial response in inflammation (assessed as IL- 6) and most blood lipids, lipid classes and subclasses. The women with RA did, however, exhibit a less pronounced response in VLDL-C, and less TGs were carried by the smallest subfraction of VLDL particles, compared to women without RA.

### Postprandial inflammation

To the best of our knowledge, the PIRA trial is the first to investigate the postprandial inflammatory response in a population with RA. Several previous studies have reported on the response to a high-fat meal in diseases with inflammatory components such as cardiovascular disease [45], asthma [46], obesity [47, 48] and metabolic syndrome [49]. Approximately twofold elevations in IL- 6 have typically been reported in both patients and controls, independent of inflammatory status at baseline [21]. When comparing the response in middle-aged men suffering premature coronary artery disease with that in healthy controls, Lundman et al. observed a 100% increase in IL- 6 at four hours after a meal in both groups [45]. Similarly, Schönknecht et al. conducted a study on older individuals with some components of metabolic syndrome and noted a 100% increase in both groups when stratified according to fasting IL- 6 concentration [49]. The increment in IL- 6 in the current study is thereby relatively large, most notably among the women without RA, where the median increase was 591%. Differences in fatty acid saturation have been shown to induce different postprandial responses in IL- 6 in some studies [50], whereas others suggest that the total energy content of the test meal may be the main predictor of the inflammatory response [47, 51]. With only just under 700 kcal and 10 E% from SFA, neither of these factors are likely to explain the large percentage increase in IL- 6 observed in our study. Previous studies have reported significantly larger postprandial increases in IL- 6 in women than in men [43, 52]. This could be a contributing factor behind the large response seen in our study population. The timing of postprandial blood sampling offers another plausible explanation. IL- 6 has typically been measured at four hours after completion of the meal, around which time peak concentrations are expected [18], whereas it was measured after five hours in our study. Based on data from the few studies with an extended follow-up time after a single meal [43, 53, 54], it is reasonable to expect that the extra hour in our study could have contributed to the more pronounced increase.

In RA, both synovial and systemic accumulation of IL- 6 is frequently observed, with higher concentrations being associated with increased disease activity and severity [55, 56]. Therefore, it was somewhat unexpected that both groups in our study exhibited similarly low fasting plasma concentrations of IL- 6. This is in contrast to earlier findings of elevated fasting levels in individuals with RA [55] and could be a reflection both of the modest disease activity in the RA group and of improvements in pharmacological treatment of the disease. While treatment with IL- 6 inhibitors was an exclusion criterion, a majority of the women were treated with methotrexate, a disease modifying drug that could influence the production of cytokines such as IL- 6 [57].

### Postprandial lipemia

Alterations in lipoprotein quantity, quality and cell cholesterol trafficking have frequently been observed in fasting blood samples from patients with treated RA and contribute to the elevated ASCVD risk associated with the disease [58]. In our study, no differences in the fasting lipid profile were observed between women with and without RA. This might be attributed to the modest disease activity, efficient pharmacological therapy, heightened awareness of ASCVD risk among patients and the fact that the PIRA control group was matched for age and BMI, which are also factors that can be expected to influence fasting blood lipid levels.

The importance of well-regulated lipid metabolism during the hours immediately after a meal has been highlighted by large population studies linking non-fasting hyperlipidemia to increased risk for ASCVD [59, 60]. In our study, the postprandial increase in TGs was close to identical in women with and without RA, and the magnitude was in line with the expectations for metabolically healthy individuals after a high-fat meal [61]. Consistent with this, in the only previously published meal trial describing the lipemic response in patients with RA, Mena-Vázquez et al. reported a similar increase in TGs after a mixed meal containing 50 g of fat and found no difference compared with matched controls [17]. Our results relating to TC, LDL-C, HDL-C, and apoB- 100 are consistent with prior studies in patients with RA and other populations that report either no change or a slight decrease after a meal [12].

Increases in VLDL-C were observed in both groups of our study, with a significantly larger overall response in women without RA. In partial agreement, Mena-Vázquez et al. reported a trend for higher postprandial VLDL-C levels in the control group but no change relative to baseline in either group [17]. The larger VLDL-C response in our study would suggest more pro-atherogenic postprandial conditions in the women without RA. After a meal, the cholesterol contained in the VLDL fraction accounts for a substantial portion of what is often categorised as remnant cholesterol or elsewhere as TRL cholesterol [62]. This cholesterol moiety is regarded as highly atherogenic due both to the large cholesterol load of each lipoprotein particle and a size that favours endothelial transcytosis, retention, and inflammation [62]. In meal studies, higher postprandial concentrations of VLDL-C have been linked to coronary artery disease [63] and carotid plaque size as a marker for atherosclerosis [64]. The significance of the slightly greater response in TGs carried in the highest density VLDL subfraction seen in the women without RA is uncertain but could indicate subtle group differences in the delipidation cascade through which TRLs are gradually catabolised after a meal. It is difficult to speculate on why this difference was found.

Numerous genetic, hormonal, and biological factors related to the assembly, secretion, clearance, and cholesterol enrichment of TRLs have been recognised to influence the postprandial lipemic response [65]. Several of these were controlled for in our study through matching criteria and statistical models, including age, BMI, habitual physical activity, and baseline plasma concentrations. Menopausal status was similar in both our groups, which is important since estrogen has been shown to exert an attenuating effect on the lipemic response [66]. Whereas overtly impaired insulin sensitivity, as evidenced by elevated HbA1c or a diagnosis of type 2 diabetes, was an exclusion criterion in the current study, no consideration was given to variations within the normal range. The link between insulin resistance and higher postprandial concentrations of TRLs has been well established [67, 68]. Proposed mechanisms include both intestinal and hepatic hypersecretion as well as delayed clearance from circulation [69, 70]. Prolonged plasma residence time is known to favour cholesterol enrichment of TRLs through increased exposure to cholesteryl ester transfer protein [65]. The significantly higher HbA1c in the women without RA in our study could indicate a lower insulin sensitivity compared with the women with RA, and this could in turn be speculated to contribute towards the observed differences in VLDL-C.

### Association between postprandial lipaemia and inflammation

Lipid metabolism and immune regulation are highly interconnected, and postprandial lipemia has been proposed as a main driver of the transient inflammatory response to a meal [52, 71]. In support of this concept, higher non-fasting TGs have been associated with low-grade, systemic inflammation in a large population study [72]. In mechanistic studies, an acute elevation of TGs has been implicated in the activation and recruitment of immune cells and production of proinflammatory cytokines contributing to endothelial dysfunction, which is regarded as an early marker for atherosclerosis [73, 74]. However, meal studies investigating the relationship between postprandial responses in TGs and IL- 6 have generally reported negative findings [45, 52, 54]. In agreement with this, our data do not support any association between these two variables.

### Strengths and limitations

The PIRA trial has several strengths. First, the test meal was carefully constructed to reflect a realistic load of calories and fat from a main meal in a typical Western diet. In combination with the mixed-meal approach, this makes the metabolic challenges representative of the ones faced by many individuals on an everyday basis. Another strength is the strict selection criteria, resulting in a well-matched control group that would have allowed us to isolate any effects related to RA. Last, the use of NMR technology provided us with detailed data from a wide range of lipid fractions, which increased our ability to identify even subtle differences in postprandial lipid metabolism. At the same time, the differences both in analytical methods and classification of lipid fractions warrant caution when comparing our results to those from other studies.

The current study is not without its limitations. The power calculation was not based on any of the outcomes reported in this paper, which means that the risk for type 2 errors should not be neglected. It is also possible that the observed differences are the result of type 1 error due to multiple testing, but this study is explorative, and the findings must be confirmed in additional studies. In designing the PIRA meal challenges, some compromises were made of respect for the participant’s comfort. A longer postprandial follow-up time and some additional samples could have increased the precision of our data. It is plausible that the chosen sampling regimen may not have captured the peak concentrations of either TGs or IL- 6 with the desired accuracy. The validity of the cytokine IL- 6 as a marker for postprandial inflammation could be debated, as it is released into circulation in almost all situations of homeostatic perturbation [75]. Pertinent to our discussion, the use of an in-dwelling cannula for serial blood sampling has been shown to elicit a local increase in IL- 6, even in the absence of food ingestion [76]. This could indeed be a factor contributing to the excessive IL- 6 response seen in a handful of individuals in each group of our study. Assessing more than one marker of postprandial inflammation would have allowed for a more nuanced representation of this highly dynamic state. Finally, the ethnic homogeneity of our sample, the all-female study participants and the modest disease activity in the RA group limit the generalizability of our findings.

## Conclusion

In conclusion, the inflammatory and lipemic responses to a high-fat meal containing red meat were largely similar in women with RA compared with matched women without RA. Hence, our data do not support a role for either postprandial inflammation or lipemia in the perceived aggravation of symptoms described by patients with RA upon consumption of red meat or in the elevated ASCVD risk associated with the disease. If anything, our findings would suggest a less pro-atherogenic postprandial condition in women with RA due to the smaller response in VLDL-C. Nevertheless, the women with RA were on satisfying pharmacological treatment, and most were in remission, which can explain why few differences were detected between the groups.

## Supplementary Information

Additional file 1

## Data Availability

The data underlying this article cannot be shared publicly considering the Swedish law on privacy of the individuals involved in the study. However, the data can be shared upon reasonable request to the corresponding author.

## Abbreviations

Apo: Apolipoprotein
ACR: American College of Rheumatology
ASCVD: Atherosclerotic Cardiovascular Disease
AUC_min_: Area Under the Curve with minimum concentration as baseline
BMI: Body Mass Index
-C: Cholesterol
CI: Confidence Interval
CM: Chylomicron
COVID: Coronavirus Disease
DAS: Disease Activity Score
E%: Energy Percentage
ECLIA: Electrochemiluminescence Immunoassay
ESC: European Society of Cardiology
ESR: Erythrocyte Sedimentation Rate
EULAR: European League Against Rheumatism
HbA1c: Glycated Haemoglobin
HDL: High Density Lipoprotein
hsCRP: High Sensitivity C-Reactive Protein
IQR: Interquartile Range
IDL: Intermediate Density Lipoprotein
IL- 6: Interleukin- 6
LDL: Low Density Lipoprotein
NMR: Nuclear Magnetic Resonance
-P: Particles
PIRA: Postprandial Inflammation in Rheumatoid Arthritis
-PL: Phospholipids
RA: Rheumatoid Arthritis
RM-ANOVA: Repeated Measures Analysis of Variance
SD: Standard Deviation
TC: Total Cholesterol
TG: Triglyceride
TRL: Triglyceride
