# Supplementary material for: Inflammatory and lipemic response to red meat intake in women with and without Rheumatoid Arthritis: a single meal study within a randomized controlled trial
Source: BMC Nutr. 2025 Apr 11;11:74. doi: 10.1186/s40795-025-01055-9 (PMC11987391; doi:10.1186/s40795-025-01055-9)
Supplement: Supplementary file 1 — Additional file 1 [file 40795_2025_1055_MOESM1_ESM.docx]

**Supplemental table S.1.** Components of the test meals

|  | Per meal (g) | Specifications | Brand |
| --- | --- | --- | --- |
|  |  |  |  |
| Minced meat | 130 | 60/40 beef/pork, 20% fat | Scan |
| Breadcrumbs | 8 |  |  |
| Whole eggs | 25 |  |  |
| Salt | 1,4 |  |  |
| White bread | 84 | “Jättefranska”, toasted | Pågens |
| Dressing | 20 | Hamburgerdressing, vegan | Rydbergs |
| Canola oil | 6 | 100% rapeseed | Zeta |
| Romaine lettuce | 10 | Fresh |  |
| Cucumber | 20 | Fresh |  |
| Tomato | 30 | Fresh |  |
|  |  |  |  |

**Supplemental table S.2**. Baseline and temporal changes in plasma lipid concentrations after the test meal

|  | Timepoint | | |  | |  | |  | |  | |  | | | Time^3^ | Group x Time^4^ |
| --- | --- | --- | --- | --- | --- | --- | --- | --- | --- | --- | --- | --- | --- | --- | --- | --- |
|  | 0 | | | Δ30 min^1^ | | Δ60 min^1^ | | Δ120 min^1^ | | Δ180 min^1^ | | Δ300 min^1^ | | |  |  |
|  | Median | | (IQR) | Median | (IQR) | Median | (IQR) | Median | (IQR) | Median | (IQR) | Median | | (IQR) | *p* | *p* |
| TG, *mmol/l* |  | | |  | |  | |  | |  | |  | | |  | 0.527 |
| Women with RA | 0.86 | | (0.41) | 0.059 | (0.12) | 0.13 | (0.27) | 0.30 | (0.25) | 0.38 | (0.20) | 0.30 | | (0.37) | <0.001* |  |
| Women without RA | 0.97 | | (0.41) | 0.020 | (0.13) | 0.23 | (0.25) | 0.29 | (0.35) | 0.44 | (0.39) | 0.27 | | (0.46) | <0.001* |  |
| *p^2^* | *0.549* | | | *0.796* | | *0.673* | | *0.639* | | *0.557* | | *0.925* | | |  |  |
| TC, *mmol/l* |  | | |  | |  | |  | |  | |  | | |  | 0.609 |
| Women with RA | 5.18 | (1.69) | | 0.049 | (1.09) | -0.041 | (1,01) | -0.047 | (0.48) | 0.15 | (0.62) | 0.035 | (0.85) | | 0.911 |  |
| Women without RA | 5.57 | (1.64) | | -0.33 | (0.71) | -0.14 | (0.76) | -0,28 | (1.10) | -0,28 | (1.19) | -0,41 | (0.67) | | 0.443 |  |
| *p^2^* | *0.205* | | | *0.888* | | *0.453* | | *0.814* | | *0.778* | | *0.690* | | |  |  |
| LDL-C, *mmol/l* |  | | |  | |  | |  | |  | |  | | |  | 0.491 |
| Women with RA | 2.95 | (1.47) | | 0.13 | (0.69) | 0.060 | (0.64) | -0.046 | (0.37) | -0.0047 | (0.42) | -0.20 | (0.61) | | 0.233 |  |
| Women without RA | 3.10 | (1.64) | | -0.17 | (0.48) | -0.12 | (0.57) | -0.23 | (0.84) | -0.25 | (0.74) | -0.41 | (0.40) | | 0.090 |  |
| *p^2^* | *0.222* | | | *0.925* | | *0.622* | | *0.814* | | *0.888* | | *0.869* | | |  |  |
| HDL-C, *mmol/l* |  | | |  | |  | |  | |  | |  | | |  | 0.562 |
| Women with RA | 1.77 | (0.45) | | 0.049 | (0.29) | 0.026 | (0.31) | 0.020 | (0.17) | 0.069 | (0.20) | 0.010 | (0.31) | | 0.542 |  |
| Women without RA | 1.76 | (0.43) | | -0.070 | (0.32) | -0.036 | (0.23) | -0.040 | (0.48) | -0.10 | (0.32) | -0.11 | (0.22) | | 0.344 |  |
| *p^2^* | *0.725* | | | *0.851* | | *0.760* | | *0.778* | | *0.656* | | *0.760* | | |  |  |
| ApoA-I, *g/l* |  | | |  | |  | |  | |  | |  | | |  | 0.639 |
| Women with RA | 1.61 | (0.29) | | -0.0062 | (0.22) | -0.033 | (0.21) | -0.042 | (0.16) | 0.012 | (0.16) | 0.030 | (0.23) | | 0.873 |  |
| Women without RA | 1.64 | (0.40) | | -0.079 | (0.26) | -0.063 | (0.17) | -0.067 | (0.35) | -0.10 | (0.26) | -0.079 | (0.18) | | 0.419 |  |
| *p^2^* | *0.398* | | | *0.944* | | *0.439* | | *0.851* | | *0.888* | | *0.869* | | |  |  |
| ApoB-100, g*/l* |  | | |  | |  | |  | |  | |  | | |  | 0.614 |
| Women with RA | 0.81 | (0.33) | | 0.022 | (0.14) | 0.010 | (0.15) | -0.0016 | (0.10) | 0.029 | (0.09) | 0.025 | (0.13) | | 0.624 |  |
| Women without RA | 0.89 | (0.31) | | -0.040 | (0.08) | -0.015 | (0.10) | -0.015 | (0.17) | -0.035 | (0.15) | -0.044 | (0.08) | | 0.430 |  |
| *p^2^* | *0.348* | | | *0.796* | | *0.796* | | *0.796* | | *0.963* | | *0.963* | | |  |  |
|  |  | | |  | |  | |  | |  | |  | | |  |  |
| VLDL-P, *nmol/l* |  | | |  | |  | |  | |  | |  | | |  | 0.124 |
| Women with RA | 98.15 | (44.61) | | 2.14 | (10.24) | 7.76 | (14.90) | 15.23 | (18.50) | 22.95 | (16.10) | 21.30 | (32.72) | | <0.001* |  |
| Women without RA | 96.29 | (57.02) | | 4.18 | (15.45) | 13.75 | (16.86) | 21.80 | (19.41) | 30.29 | (25.30) | 29.28 | (31.62) | | <0.001* |  |
| *p^2^* | *0.963* | | | *0.963* | | *0.981* | | *0.526* | | *0.481* | | *0.573* | | |  |  |
| IDL-P, *nmol/l* |  | | |  | |  | |  | |  | |  | | |  | 0.466 |
| Women with RA | 60.77 | (55.77) | | -2.38 | (32.58) | -8.86 | (34.88) | -8.49 | (24.98) | -0.50 | (26.25) | 2.28 | (29.11) | | 0.076 |  |
| Women without RA | 59.93 | (40.73) | | -13.75 | (15.04) | -11.13 | (18.20) | -858 | (34.49) | -6.40 | (26.64) | -3.36 | (23.04) | | 0.015* |  |
| *p^2^* | *0.639* | | | *0.146* | | *0.944* | | *0.385.* | | *0.851* | | *0.760* | | |  |  |
| LDL-P, *nmol/l* |  | | |  | | *.* | |  | |  | |  | | |  | 0.528 |
| Women with RA | 1376.55 | (497.96) | | 43.03 | (259.66) | 2.50 | (250.33) | -26.95 | (183.96) | 3.45 | (156.57) | -32.78 | (232.72) | | 0.600 |  |
| Women without RA | 1404.37 | (527,69) | | -80.79 | (161.63) | -40.67 | (212.88) | -35.55 | (307.37) | -95.43 | (312.57) | -124.10 | (189.70) | | 0.215 |  |
| *p^2^* | *0.467* | | | *0.814* | | *0.690* | | *0.796* | | *0.851* | | *0.888* | | |  |  |
|  |  | | |  | |  | |  | |  | |  | | |  |  |
| VLDL-TG, *mmol/l* |  | | |  | |  | |  | |  | |  | | |  | 0.584 |
| Women with RA | 0.45 | (0.29) | | 0.65 | (0.07) | 0.073 | (0.14) | 0.16 | (0.19) | 0.28 | (0.12) | 0.22 | (0.29) | | <0.001* |  |
| Women without RA | 0.47 | (0.30) | | -0.0029 | (0.13) | 0.15 | (0.19) | 0.17 | (0.23) | 0.33 | (0.32) | 0.18 | (0.30) | | <0.001* |  |
| *p^2^* | *0.511* | | | *0.851* | | *0.439* | | *0.439* | | *0.425* | | *0.851* | | |  |  |
| IDL-TG, *mmol/l* |  | | |  | |  | |  | |  | |  | | |  | 0.515 |
| Women with RA | 0.050 | (0.06) | | 0.015 | (0.02) | 0.028 | (0.05) | 0.049 | (0.04) | 0.067 | (0.03) | 0.045 | (0.07) | | <0.001* |  |
| Women without RA | 0.066 | (0.06) | | 0.0029 | (0.03) | 0.035 | (0.05) | 0.045 | (0.07) | 0.075 | (0.06) | 0.041 | (0.07) | | <0.001* |  |
| *p^2^* | *0.193* | | | *0.439* | | *0.342* | | *0.360* | | *0.481* | | *0.869* | | |  |  |
| LDL-TG *mmol/l* |  | | |  | |  | |  | |  | |  | | |  | 0.876 |
| Women with RA | 0.21 | (0.08) | | -0.097 | (0.04) | -0.011 | (0.04) | -0.013 | (0.04) | -0.011 | (0.03) | -0.0077 | (0.05) | | 0.106 |  |
| Women without RA | 0.22 | (0.07) | | -0.017 | (0.02) | -0.012 | (0.03) | -0.021 | (0.04) | -0.020 | (0.05) | -0.019 | (0.03) | | 0.053 |  |
| *p^2^* | *0.656* | | | *0.213* | | *0.534* | | *0.260* | | *0.291* | | *0.260* | | |  |  |
| HDL-TG, *mmol/l* |  | | |  | |  | |  | |  | |  | | |  | 0.832 |
| Women with RA | 0.13 | (0.05) | | -0.046 | (0.02) | -0.0011 | (0.02) | 0.0046 | (0.02) | 0.016 | (0.02) | 0.016 | (0.02) | | <0.001* |  |
| Women without RA | 0.12 | (0.05) | | -0.0064 | (0.01) | 0.0016 | (0.02) | 0.0077 | (0.03) | 0.015 | (0.02) | 0.019 | (0.02) | | <0.001* |  |
| *p^2^* | *0.851* | | | *0.565* | | *0.647* | | *0.664* | | *0.907* | | *0.778* | | |  |  |
|  |  | | |  | |  | |  | |  | |  | | |  |  |
| VLDL-C, *mmol/l* |  | | |  | |  | |  | |  | |  | | |  | 0.227 |
| Women with RA | 0.26 | (0.16) | | 0.0094 | (0.05) | 0.0264 | (0.05) | 0.060 | (0.07) | 0.11 | (0.06) | 0.090 | (0.13) | | <0.001* |  |
| Women without RA | 0.24 | (0.18) | | 0.0053 | (0.07) | 0.045 | (0.07) | 0.086 | (0.11) | 0.13 | (0.10) | 0.13 | (0.16) | | <0.001* |  |
| *p^2^* | *0.869* | | | *0.760* | | *0.963* | | *0.639* | | *0.760* | | *0.639* | | |  |  |
| VLDL-FC, *mmol/l* |  | | |  | |  | |  | |  | |  | | |  | 0.245 |
| Women with RA | 0.14 | (0.05) | | 0.0035 | (0.02) | 0.013 | (0.03) | 0.025 | (0.03) | 0.043 | (0.03) | 0.042 | (0.06) | | <0.001* |  |
| Women without RA | 0.15 | (0.09) | | 0.0037 | (0.03) | 0.019 | (0.03) | 0.032 | (0.05) | 0.053 | (0.04) | 0.060 | (0.07) | | <0.001* |  |
| *p^2^* | *0.681* | | | *0.742* | | *0.805* | | *0.496* | | *0.549* | | *0.622* | | |  |  |
| VLDL-PL, *g/l* |  | | |  | |  | |  | |  | |  | | |  | 0.278 |
| Women with RA | 0.13 | (0.06) | | 0.0015 | (0.01) | 0.0069 | (0.02) | 0.015 | (0.02) | 0.035 | (0.02) | 0.034 | (0.05) | | <0.001* |  |
| Women without RA | 0.13 | (0.08) | | -0.0025 | (0.03) | 0.0163 | (0.03) | 0.030 | (0.04) | 0.047 | (0.04) | 0.045 | (0.06) | | <0.001* |  |
| *p^2^* | *0.842* | | | *0.981* | | *0.869* | | *0.496* | | *0.542* | | *0.606* | | |  |  |
|  |  | | |  | |  | |  | |  | |  | | |  |  |
| VLDL-1-TG, *mmol/l* |  | | |  | |  | |  | |  | |  | | |  | 0.310 |
| Women with RA | 0.18 | (0.17) | | 0.044 | (0.04) | 0.077 | (0.10) | 0.17 | (0.13) | 0.23 | (0.11) | 0.17 | (0.18) | | <0.001* |  |
| Women without RA | 0.23 | (0.21) | | 0.024 | (0.08) | 0.14 | (0.14) | 0.17 | (0.15) | 0.27 | (0.19) | 0.15 | (0.19) | | <0.001* |  |
| *p^2^* | *0.411* | | | *0.787* | | *0.250* | | *0.385* | | *0.360* | | *0.787* | | |  |  |
| VLDL-2-TG, *mmol/l* |  | | |  | |  | |  | |  | |  | | |  | 0.726 |
| Women with RA | 0.069 | (0.04) | | 0.013 | (0.02) | 0.020 | (0.03) | 0.026 | (0.04) | 0.034 | (0.03) | 0.023 | (0.05) | | 0.005* |  |
| Women without RA | 0.070 | (0.05) | | 0.0081 | (0.03) | 0.029 | (0.04) | 0.034 | (0.05) | 0.043 | (0.05) | 0.017 | (0.05) | | <0.001* |  |
| *p^2^* | *0,681* | | | *0,869* | | *0,467* | | *0,725* | | *0,589* | | *0,833* | | |  |  |
| VLDL-3-TG, *mmol/l* |  | | |  | |  | |  | |  | |  | | |  | 0.564 |
| Women with RA | 0.060 | (0.04) | | 0.0038 | (0.02) | 0.0095 | (0.02) | 0.0053 | (0.04) | 0.015 | (0.03) | 0.012 | (0.04) | | 0.169 |  |
| Women without RA | 0.057 | (0.05) | | -0.0001 | (0.02) | 0.010 | (0.03) | 0.019 | (0.05) | 0.022 | (0.06) | 0.0044 | (0.05) | | 0.011* |  |
| *p^2^* | *0.888* | | | *0.981* | | *0.734* | | *0.664* | | *0.690* | | *0.656* | | |  |  |
| VLDL-4-TG, *mmol/l* |  | | |  | |  | |  | |  | |  | | |  | 0.217 |
| Women with RA | 0.070 | (0.04) | | -0.0014 | (0.01) | -0.020 | (0.01) | -0.0033 | (0.02) | -0.0017 | (0.01) | 0.0067 | (0.02) | | 0.167 |  |
| Women without RA | 0.063 | (0.03) | | -0.0040 | (0.01) | -0.0029 | (0.01) | -0.0046 | (0.03) | 0.0034 | (0.02) | 0.0102 | (0.02) | | <0.001* |  |
| *p^2^* | *0.814* | | | *0.622* | | *0.622* | | *0.869* | | *0.944* | | *0.481* | | |  |  |
| VLDL-5-TG, *mmol/l* |  | | |  | |  | |  | |  | |  | | |  | 0.220 |
| Women with RA | 0.031 | (0.01) | | -0,0005 | (0.01) | -0.0006 | (0.01) | 0.0020 | (0.01) | 0.0019 | (0.00) | 0.0033 | (0.01) | | 0.038* |  |
| Women without RA | 0.030 | (0.01) | | 0,0030 | (0.01) | -0.0001 | (0.01) | 0.0019 | (0.01) | 0.0051 | (0.01) | 0.0082 | (0.01) | | <0.001* |  |
| *p^2^* | *0.639* | | | *0.690* | | *0.851* | | *0.981* | | *0.342* | | *0.255* | | |  |  |
|  |  | | |  | |  | |  | |  | |  | | |  |  |

Apo, Apolipoprotein ; -C, Cholesterol ; -FC, Free cholesterol ; HDL, High density lipoprotein ; IDL, Intermediate density lipoprotein ; LDL, Low density lipoprotein ; -P, Particles ; -PL, Phospholipids ; RA, Rheumatoid arthritis ; TC, Total cholesterol ; TG, Triglycerides ; VLDL, Very-low density lipoprotein

*Significant at the α-level 0.05

^1^Δ denotes the difference in plasma concentrations compared with baseline (t0)

^2^Group medians of absolute values compared at each timepoint using independent samples t-test.

^3^One-way repeated measures ANOVA with plasma concentrations at timepoint t0-t5 as within subjects variables.

^4^Two-way repeated measures ANOVA with plasma concentrations at timepoint t0-t5 as within subjects variables and group affiliation as between subjects factor

**Supplemental table S.3.** Area under the curve with minimum concentration as baseline (AUC_min_) for selected lipid parameters

|  | Women with RA  (n=22) | |  | Women without RA  (n=22) | |  | *p^1^* | *p^2^* |
| --- | --- | --- | --- | --- | --- | --- | --- | --- |
|  | Median | IQR |  | Median | IQR |  |  |  |
|  |  |  |  |  |  |  |  |  |
| TG, *mmol/l · 300 min* | 100.38 | 73.10-110.96 |  | 98.40 | 63.09-126.42 |  | 0.542 | 0.592 |
|  |  |  |  |  |  |  |  |  |
| Cholesterol and apolipoproteins |  |  |  |  |  |  |  |  |
| TC, *mmol/l · 300 min* | 133.03 | 85.45-246.27 |  | 194.79 | 96.27-487.91 |  | 0.159 | 0.184 |
| LDL-C,  *mmol/l · 300 min* | 92.92 | 65.24-172.76 |  | 145.93 | 85.50-301.81 |  | 0.159 | 0.291 |
| HDL-C,  *mmol/l · 300 min* | 41.21 | 23.52-84.04 |  | 60.32 | 38.28-113.65 |  | 0.181 | 0.273 |
| ApoA-I, *g/l · 300 min* | 33.97 | 18.70-64.49 |  | 48.45 | 21.21-105.48 |  | 0.166 | 0.280 |
| ApoB-100, g*/l · 300 min* | 18.68 | 11.97-34.27 |  | 28.54 | 11.97-61.64 |  | 0.324 | 0.490 |
|  |  |  |  |  |  |  |  |  |
| ApoB-carrying lipoprotein particles, main fractions | | |  |  |  |  |  |  |
| VLDL-P, *nmol/l · 300 min ∙10^-3^* | 5.91 | 3.43-7.66 |  | 7.11 | 4.69-9.78 |  | 0.064 | 0.076 |
| IDL-P, *mmol/l · 300 min ∙10^-3^* | 4.03 | 2.59-5.38 |  | 4.35 | 2.18-9.85 |  | 0.110 | 0.142 |
| LDL-P, *nmol/l · 300 min ∙10^-3^* | 40.11 | 20.18-62.82 |  | 49.94 | 30.60-113.12 |  | 0.241 | 0.329 |
|  |  |  |  |  |  |  |  |  |
| TG-distribution between lipoprotein main fractions | | |  |  |  |  |  |  |
| VLDL-TG, *mmol/l · 300 min* | 66.90 | 50.47-77.86 |  | 75.35 | 42.82-87.41 |  | 0.489 | 0.838 |
| IDL-TG, *mmol/l · 300 min* | 18.17 | 12.22-19.54 |  | 17.55 | 9.88-20.77 |  | 0.925 | 0.516 |
| LDL-TG, *mmol/l · 300 min* | 4.67 | 2.11-8.61 |  | 7.62 | 3.96-14.29 |  | 0.035* | 0.344 |
| HDL-TG, *mmol/l · 300 min* | 4.69 | 3.51-6.19 |  | 5.80 | 3.88-7.31 |  | 0.222 | 0.431 |
|  |  |  |  |  |  |  |  |  |
| VLDL-composition | | |  |  |  |  |  |  |
| VLDL-C, *mmol/l · 300 min* | 24.08 | 17.45-32.50 |  | 30.27 | 22.87-40.32 |  | 0.116 | 0.028* |
| VLDL-FC, *mmol/l · 300 min* | 10.27 | 7.41-13.72 |  | 14.01 | 9.00-16.90 |  | 0.091 | 0.080 |
| VLDL-PL, *g/l · 300 min* | 8.89 | 5.81-10.63 |  | 11.91 | 7.23-15.09 |  | 0.046* | 0.068 |
|  |  |  |  |  |  |  |  |  |
| TG-distribution between VLDL-subfractions | | |  |  |  |  |  |  |
| VLDL-1-TG  *mmol/l · 300 min* | 55.04 | 43.97-63.80 |  | 53.20 | 33.52-72.59 |  | 0.963 | 0.958 |
| VLDL-2-TG  *mmol/l · 300 min* | 9.11 | 7.20-11.78 |  | 11.10 | 6.83-15.94 |  | 0.296 | 0.370 |
| VLDL-3-TG  *mmol/l · 300 min* | 6.38 | 3.71-8.55 |  | 8.45 | 4.23-12.78 |  | 0.121 | 0.130 |
| VLDL-4-TG  *mmol/l · 300 min* | 2.22 | 1.42-5.15 |  | 3.61 | 2.63-6.06 |  | 0.082 | 0.065 |
| VLDL-5-TG  *mmol/l · 300 min* | 1.28 | 0.62-2.11 |  | 1.65 | 1.29-2.53 |  | 0.049* | 0.028* |
|  |  |  |  |  |  |  |  |  |

Apo, Apolipoprotein ; -C ; Cholesterol ; -FC, Free cholesterol ; HDL, High density lipoprotein ; IDL, Intermediate density lipoprotein ; LDL; Low density lipoprotein ; -PL, Phospholipids ; RA, Rheumatoid arthritis ; TG, Triglycerides ; TC, Total cholesterol ; VLDL, Very-low density lipoprotein

*Significant at the α-level 0.05

^1^Group medians compared using Mann-Whitney U-test

^2^Group differences from multivariable linear regression model, with baseline plasma concentration, age, body mass index and physical activity level as covariates. Outcome variables log10-transformed as needed to fit model assumptions.

**Supplemental table S.4.** Multivariable regression of the effect of postprandial triglyceride-response on the change in interleukin-6

| Dependent variable | | Independent variables | β^a^ | 95% CI | *p* |
| --- | --- | --- | --- | --- | --- |
|  | |  |  |  |  |
| $∛$(Percentual increase in IL-6) | | $∛$(AUC_min_-TG) | -1.13 | -3.41 ; 1.16 | 0.324 |
|  | | IL-6 concentration before meal | -1.95 | -3.16 ; -0.75 | 0.002 |
|  | | Age | 0.034 | -0.20 ; 0.27 | 0.770 |
|  | | Body mass index | -0.12 | -0.64 ; 0.41 | 0.653 |
|  | | Physical activity | -0.23 | -1.79 ; 1.33 | 0.767 |
| R^2^ | 0.279 |  |  |  |  |
| F-test | 2.713 *p*=0.036 |  |  |  |  |
| *n^b^*= | 41 |  |  |  |  |
|  | |  |  |  |  |

AUC_min_, Area under the curve minimum concentration; CI, Confidence interval ; IL-6, Interleukin-6

^a^Transformed numbers

^b^Three individuals from RA-group had to be excluded to fit model assumptions.
